# Over-projected Pacific warming and extreme El Niño frequency due to CMIP5 common biases

**DOI:** 10.1093/nsr/nwab056

**Published:** 2021-04-06

**Authors:** Tao Tang, Jing-Jia Luo, Ke Peng, Li Qi, Shaolei Tang

**Affiliations:** Institute for Climate and Application Research (ICAR), Nanjing University of Information Science and Technology, Nanjing 210044, China; Key Laboratory of Meteorological Disaster of Ministry of Education/Joint International Research Laboratory of Climate and Environment Change/Collaborative Innovation Center on Forecast and Evaluation of Meteorological Disasters, Nanjing University of Information Science and Technology, Nanjing 210044, China; Institute for Climate and Application Research (ICAR), Nanjing University of Information Science and Technology, Nanjing 210044, China; Key Laboratory of Meteorological Disaster of Ministry of Education/Joint International Research Laboratory of Climate and Environment Change/Collaborative Innovation Center on Forecast and Evaluation of Meteorological Disasters, Nanjing University of Information Science and Technology, Nanjing 210044, China; Institute for Climate and Application Research (ICAR), Nanjing University of Information Science and Technology, Nanjing 210044, China; Key Laboratory of Meteorological Disaster of Ministry of Education/Joint International Research Laboratory of Climate and Environment Change/Collaborative Innovation Center on Forecast and Evaluation of Meteorological Disasters, Nanjing University of Information Science and Technology, Nanjing 210044, China; Key Laboratory of Meteorological Disaster of Ministry of Education/Joint International Research Laboratory of Climate and Environment Change/Collaborative Innovation Center on Forecast and Evaluation of Meteorological Disasters, Nanjing University of Information Science and Technology, Nanjing 210044, China; Institute for Climate and Application Research (ICAR), Nanjing University of Information Science and Technology, Nanjing 210044, China

**Keywords:** common model biases, Pacific SST change projection, extreme El Niño frequency change, global warming, emergent constraint method

## Abstract

Extreme El Niño events severely disrupt the global climate, causing pronounced socio-economic losses. A prevailing view is that extreme El Niño events, defined by total precipitation or convection in the Niño3 area, will increase 2-fold in the future. However, this projected change was drawn without removing the potential impacts of Coupled Model Intercomparison Project phase 5 (CMIP5) models’ common biases. Here, we find that the models’ systematic biases in simulating tropical climate change over the past century can reduce the reliability of the projected change in the Pacific sea surface temperature (SST) and its related extreme El Niño frequency. The projected Pacific SST change, after removing the impacts of 13 common biases, displays a ‘La Niña-like’ rather than ‘El Niño-like’ change. Consequently, the extreme El Niño frequency, which is highly linked to the zonal distribution of the Pacific SST change, would remain mostly unchanged under CMIP5 warming scenarios. This finding increases confidence in coping with climate risks associated with global warming.

## Teaser

In stark contrast to the prevailing view of a 2-fold increase, this study suggests that extreme El Niño frequency would remain almost unchanged under global warming, highlighting the great impacts of models’ common biases on future projection.

## INTRODUCTION

An extreme El Niño event, characterized by massive warm sea surface temperature (SST) anomalies (SSTAs) and strong convection in the eastern Pacific, can severely impact the climate, agriculture, economy, marine ecosystems and environment worldwide [1–9]. It is therefore of great importance to improve the projection of future extreme El Niño frequency change under global warming [2–4,7–9].

According to Coupled Model Intercomparison Project phase 5 (CMIP5) projections [10], the Walker circulation would weaken because of the increased atmospheric static stability under global warming [7,11]. The weakened Walker circulation, in turn, would lead to an ‘El Niño-like’ SST change in the tropical Pacific, and enhanced convection and rainfall in the Niño3 region (Fig. S1 in the online Supplementary Data). Since the extreme El Niño is defined by the Niño3 total precipitation/vertical velocity [2–4], the 2-fold increase of the projected extreme El Niño frequency in the future is closely linked to this background change. However, there is a great uncertainty in the El Niño-like SST change projection [5,8,12,13].

**Figure 1.**
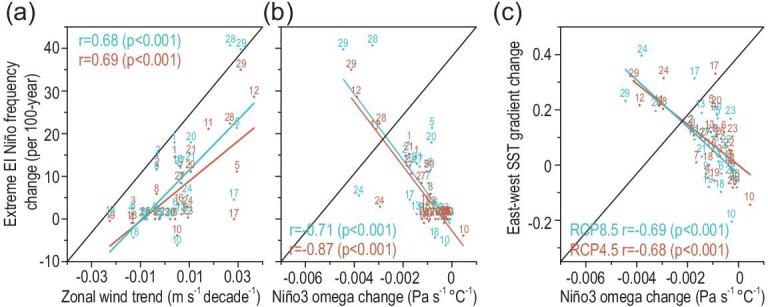
Inter-model correlations among the extreme El Niño frequency change, process simulation and mean-state changes in the CMIP5 models. (a) Inter-model correlation between the simulated trends of the zonal wind in the western-central Pacific (150°E–150°W, 5°S–5°N) during 1901–2010 and the projected extreme El Niño frequency changes in the future (2011–2098 vs. 1901–2010, in events per 100 years). Extreme El Niño is defined by the Niño3 total omega (Pa s^–1^) averaged from 500 hPa to 0 hPa, being negative (i.e. convection) in boreal winter (DJF) [4]. Correlation coefficients and p-values are indicated in each panel. The results of RCP4.5 and RCP8.5 are represented by red and blue, respectively. (b and c) Inter-model correlation of the projected Niño3 omega changes with the projected extreme El Niño frequency changes and the Pacific east-minus-west SST gradient changes in the future, respectively.

A remarkable controversy of the Pacific SST change between the CMIP5 simulations and observations of 1981–2010 has been highlighted recently [12,14–18]. Specifically, all CMIP5 models reproduce a spurious warming in the eastern Pacific, in stark contrast to the cooling and intensified Walker circulation in observations [12,14–21]. This common failure may reduce the credibility of the projected El Niño-like SST change in the future. In addition to possible influences of the internal decadal variability (i.e. Pacific Decadal Oscillation (PDO) or Interdecadal Pacific Oscillation (IPO) [19,20], it was found that the CMIP5 models’ ability to simulate the recent Pacific SST cooling is reduced by nearly 50% due to common model biases [14]. Here, we find that the CMIP5 biases in simulating 13 well-recognized processes/mean-states can largely affect the projected extreme El Niño frequency change in the future (Fig. 1a, Fig. S2 and Table S1). Biases in these processes were suggested to have noticeable impacts on the simulations of the tropical Pacific SST [12–18,22–27].

In particular, the CMIP5 experiments display significant inter-model relations among the simulated zonal wind trend in the central-western Pacific, the Pacific east-minus-west SST gradient trend and cold tongue mean-state over the past century and the projected extreme El Niño frequency change in the future (Fig. 1a and Fig. S2b and f). Models that produce stronger easterly wind trend, weaker warming in the eastern Pacific and colder cold tongue in the 20th century would project less increase or even the decrease of extreme El Niño frequency in the 21st century. The inter-model relations between the other 10 well-known processes/mean-states and the projected change in the extreme El Niño frequency vary among them (Fig. S2). Based on a multiple linear regression model with the 13 processes/mean-states as predictors (see Supplementary Data), we find the correlation between the originally projected and the reconstructed extreme El Niño frequency change reaches 0.92 and 0.90 in the RCP4.5 and RCP8.5 (Representative Concentration Pathway) scenarios, respectively (Table S2).

Since the extreme El Niño is defined by the total omega value (i.e. the sum of the mean-state and anomaly) [2–4], the projected change in the extreme El Niño frequency is therefore determined by either mean-state change or anomaly change or both. Our results indicate the multi-model mean of the CMIP5 models project nearly unchanged SST anomaly variance in the eastern Pacific (Fig. S3a–c), albeit with an insignificant increase in the central Pacific. While the geographical centers of El Niño-Southern Oscillation (ENSO) may slightly shift among the CMIP5 models [9], the maximum SST anomaly variance mostly stays in the Niño3 area (Fig. S3d). In addition, the relation between the SST anomaly forcing and the vertical velocity (omega) anomaly in the eastern Pacific would not change much in the future (Fig. S3e). Therefore, the future change in the extreme El Niño frequency defined by Niño3 total omega [4] does not appear to be determined by the anomaly change in the CMIP5 projections.

Exploratory analyses indicate the projected change in the extreme El Niño frequency is highly correlated with the change in Niño3 omega mean-state (i.e. the eastern branch of the basin-wide Walker circulation, Fig. 1b). Their inter-model correlation reaches −0.87 and −0.71 in the RCP4.5 and RCP8.5 scenario, respectively. Moreover, the Niño3 omega mean-state change is closely linked to the change in the Pacific east-minus-west SST gradient (Fig. 1c) [28].

Concerning the deterministic role of the mean-state changes in the extreme El Niño frequency change and the large impacts of the model biases on past and future climate simulations [12–18,22–27], our findings highlight the importance of a proper correction of the impacts of the models’ common biases. This is crucial to producing a more reliable projection of the extreme El Niño frequency change in the future.

## RESULTS

### Over-projected El Niño-like warming in the Pacific

Based on 30 CMIP5 coupled models (Table S3), we examine the impacts of common biases in the 13 well-recognized processes/mean-states in the present-day period (1901–2010), which includes those that are externally forced and internally induced, on the projections of future Pacific climate change (2011–2098). We select the 110-year period to minimize possible influences of internal decadal variability (e.g. PDO or IPO). Results based on 88 years (1923–2010) and recent decades with high-quality observations (1981–2010) are similar.

**Figure 2.**
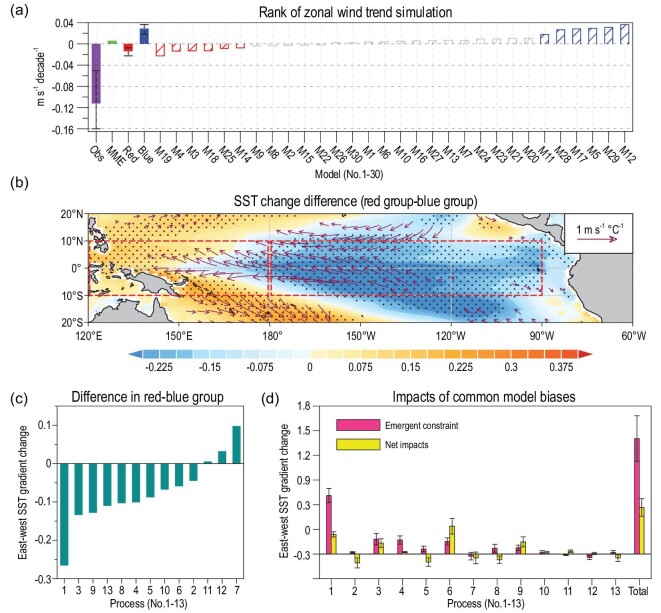
Impacts of 13 common biases of CMIP5 models on the projected SST change in the Pacific. (a) Rank of the simulated zonal wind trends in the central-western Pacific (150°E–150°W, 10°S–10°N) during 1901–2010. Filled purple, green, red and blue bars denote the easterly wind trend in observation, CMIP5 MME, red group mean and blue group mean, respectively. The error bar in (a) represents the range from the lowest to the highest values in the observation, red and blue group. (b) Differences (i.e. red group minus blue group) in the projected SST and surface wind change (i.e. 2011–2098 minus 1901–2010, divided by the global mean SST change). Stippling and vector indicate statistical confidence at the 90% level according to student *t*-test. (c) Differences in the Pacific east-minus-west SST gradient change between the red and blue groups, estimated for each process based on the results shown in Figs 2b and 3, and Fig. S4. (d) Impacts of the common biases on the MME projections of the Pacific east-minus-west SST gradient change. Pink bars denote the impacts of individual common biases, calculated by emergent constraint method. Yellow bars denote the net impacts of these common biases. The error bars in (d) represent ±1 standard deviation. Detailed discussions of the model grouping, emergent constraint and the multiple linear regression methods are shown in the Supplementary Data.

The CMIP5 models display large systematic biases in reproducing the centennial trends of the zonal wind in the central-western Pacific, the cold tongue SST, and the Pacific east-minus-west SST gradient (Fig. 2a and Fig. S4a and c). Consistent with the Pacific thermostat mechanism and Bjerknes feedback [5,6], the latter two processes are significantly correlated with the first process (Table S4). Therefore, biases in the three processes exert a similar impact. Our results indicate that models with better simulations of the three centennial trends (i.e. the red groups) tend to project a weaker east-minus-west SST warming gradient in the tropical Pacific during 2011–2098 (Fig. 2b and c). Each of the three CMIP5 common biases, calculated by the differences between the multi-model ensemble mean (MME) simulations and the observations over the past century, would lead to an over-projected east-minus-west SST gradient in the Pacific in the future (Fig. 2d).

**Figure 3.**
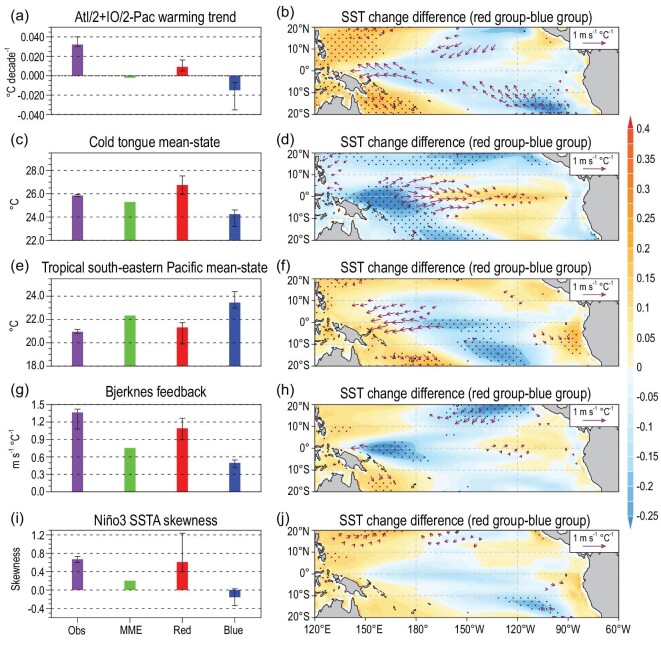
(a–j) Five biases in simulating the present-day climate and their inter-group differences on the future projection. As in Fig. 2a and b, but the results for the Atl/2 + IO/2-Pac SST warming trend, cold tongue mean-state, tropical south-eastern Pacific mean-state, Bjerknes feedback and Niño3 SSTA skewness, respectively (Table S1).

Recent studies suggested that the faster SST warming in the Atlantic and Indian Ocean than that in the Pacific over the past few decades/century can also induce the easterly trend in the central-western Pacific via modifying the Walker circulation, and hence decreasing the SST in the eastern Pacific [12,14–18]. The SST in the Indian Ocean and the Atlantic would keep warming under the increasing greenhouse gases, and hence continuously exert influences on Pacific climate change in the future. Accordingly, the models that better reproduce this inter-basin mechanism during 1901–2010 (Fig. 3a) would project a weaker east-minus-west SST warming gradient in the tropical Pacific during 2011–2098 (Figs 2c and 3b).

Similarly, the CMIP5 models also underestimate the tropical inter-basin decadal coupling among the Pacific, Atlantic and Indian oceans (Fig. S4e and g). Both the Atlantic–Pacific coupling and the Indian Ocean–Pacific coupling display a close relation to Pacific decadal climate change, indicating the importance of pan-tropical interactions [12,14–18]. Models with stronger decadal inter-basin couplings tend to project a weaker SST warming in the central-eastern tropical Pacific (Fig. 2c) [12]. Our results indicate that the commonly underestimated centennial/decadal inter-basin couplings in CMIP5 models would lead to an over-projected east-minus-west SST gradient in the Pacific (Fig. 2d).

The CMIP5 models also display long-standing biases in reproducing climatology in the tropics [22]. One well-known mean-state bias is that the Pacific cold tongue is too cold (Fig. 3c). The colder and westward-extended cold tongue tends to suppress deep convection, increasing surface insolation and hence inducing stronger warming in the central-western Pacific [24,26], and *vice versa* for a warmer cold tongue bias (Figs 2c and 3d). In agreement with previous studies [24,26], the CMIP5 common bias with a colder cold tongue would result in an under-projected east-minus-west SST gradient in the Pacific (Fig. 2d).

Other long-standing mean-state biases include the warm SST biases in the tropical south-eastern Pacific [14,22] and south-eastern Atlantic [16,22] (Fig. 3e and Fig. S4i), possibly owing to the errors in simulating the coastal upwelling and SST-stratus cloud feedback there [14,22]. The two warm biases could reduce the Pacific trade winds via modifying the Walker circulation [16]. Consistently, models that better simulate the SST mean-states in the two coastal regions tend to project stronger SST warming in the western Pacific than in the east (Figs 2c and 3f, and Fig. S4j). Thus, the CMIP5 MME warm biases in the tropical south-eastern Pacific and south-eastern Atlantic would generate an over-projected east-minus-west SST gradient in the Pacific (Fig. 2d).

It has been well recognized that interannual SST anomalies in the tropical Atlantic, Indian and Pacific oceans can also influence one another via atmosphere and ocean bridges [12,14,17,29–31]. While the influences of ENSO on the Indian Ocean and Atlantic SSTs are well simulated in most CMIP5 models [14], the influences of the Indian Ocean and Atlantic on ENSO are underestimated (cf. green and purple bars in Fig. S4k). Because underlying mechanisms of inter-basin interactions on interannual timescales are similar to those on decadal timescales [12,14,17], the systematically underestimated interannual inter-basin influence in the CMIP5 MME would similarly lead to a weak over-projection of the tropical Pacific east-minus-west SST gradient in the future (Fig. 2d).

Active ocean–atmosphere feedback in the tropical Pacific generates the strongest year-to-year climate variability (i.e. ENSO) on Earth [1]. Biases in reproducing the ENSO-related ocean–atmosphere feedback might also affect the projections of future climate change. Bjerknes feedback, measured by the regression coefficient of Niño4 zonal wind anomaly onto Niño3 SST anomaly, plays a major role in ENSO growth [23,32]. The Bjerknes feedback is systematically underestimated in CMIP5 models [23] (Fig. 3g). Models with better Bjerknes feedback project a weak increase of the Pacific east-minus-west SST gradient in the future (Figs 2c and 3h). The SST-cloud feedback in the cold tongue region provides a major damping for ENSO [14,23,32], which is also underestimated in the CMIP5 MME (Fig. S4m). Surprisingly, models with better atmospheric damping project a stronger east-minus-west SST warming gradient (Fig. 2c). This is possibly because the models with a stronger SST-cloud feedback also display stronger Bjerknes feedback (i.e. error compensation) and a warmer cold tongue mean-state, as indicated by the significant correlations among them. Thus, the CMIP5 common biases in the three processes/mean-state would lead to a similar under-projection of the east-minus-west SST gradient in the Pacific (Fig. 2d).

In addition, the asymmetry between El Niño and La Niña, represented by the positive Niño3 SST anomaly skewness, can also induce decadal mean-state change in the Pacific [13,27]. However, the skewness is systematically underestimated or even negative in the CMIP5 models (Fig. 3i). Our result indicates that models with realistic skewness tend to project a ‘La Niña-like’ SST change, except the warming along the western coast of South America [13,27] (Figs 2c and 3j). Correspondingly, the common bias with an underestimated skewness would lead to an over-projected east-minus-west SST warming gradient in the Pacific in the future (Fig. 2d).

**Figure 4.**
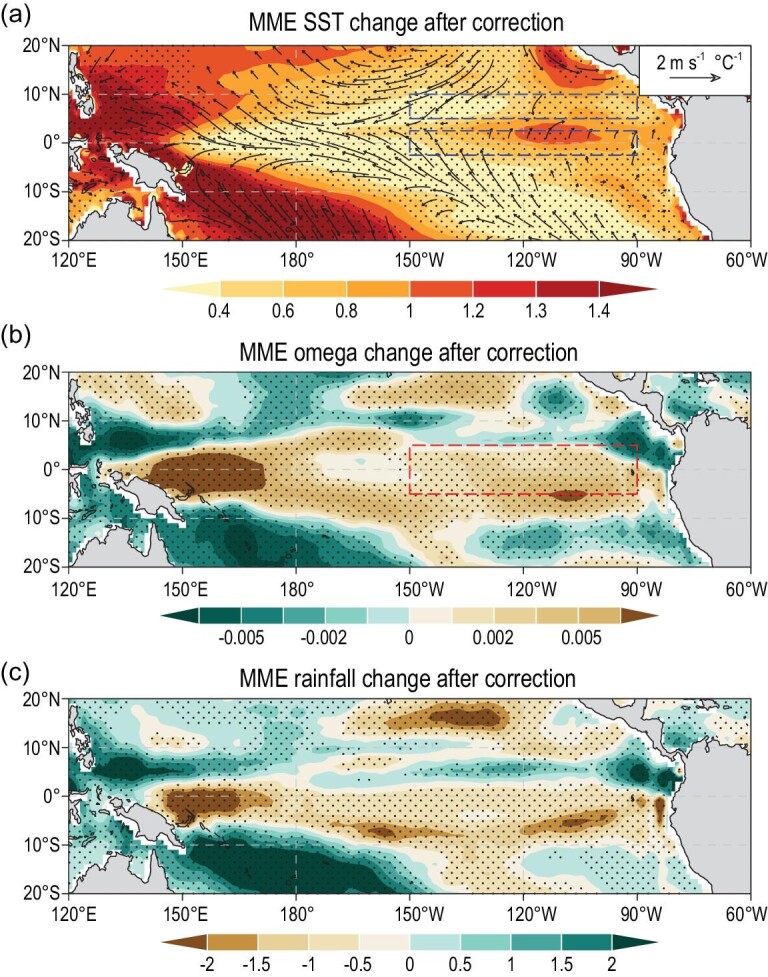
MME-projected future changes in the tropical Pacific after the correction. (a) MME projected SST and wind changes after removing the total net impacts of 13 common biases of 30 CMIP5 models. The SST and wind change refer to the difference between 2011–2098 and 1901–2010, normalized by global mean SST change in each model before calculating the MME. The two dashed blue boxes are used to calculate the north-minus-south meridional SST gradient [2–4] shown in Fig. 5. Stippling and vector indicate more than 75% of the CMIP5 models agree with the sign of the MME-projected SST and zonal wind change, respectively. (b) As in (a), but for the omega changes. Units: Pa s^–1^ °C^–1^. Dashed red box indicates the Niño3 region. (c) As in (a), but for the rainfall change. Units: mm day^–1^ °C^–1^.

A simple summation of the impacts of the 13 common biases on the future projected Pacific east-minus-west SST gradient change would reach 1.3°C per degree of global SST warming (Fig. 2d). However, many of these biases are significantly correlated with one another (Table S4). After removing their interdependent impacts with a multiple linear regression method, the net impacts of individual common biases differ largely (Fig. 2d). The total net impacts of the 13 common biases indicate that the Pacific east-minus-west SST gradient change would be over-projected by ∼0.52°C per degree of global SST warming in the future (Fig. 2d). More importantly, the CMIP5 MME projection of the Pacific SST change after the correction would no longer resemble the original El Niño-like pattern. Instead, the corrected projection displays a La Niña-like SST change with weaker warming in the east than in the west and intensified trade winds in the Pacific (cf. Fig. 4a and Fig. S1a) [28].

Consistent with the close relation between the Niño3 omega change and the Pacific east-minus-west SST gradient change, the MME projected omega change, after removing the total net impacts of the 13 common biases, displays an enhanced subsidence in the equatorial Pacific (Fig. 4b). Consistently, the projected precipitation change after the correction also displays a reduction in the equatorial Pacific (Fig. 4c). These are opposite to the original projections.

### Over-projected extreme El Niño frequency

**Figure 5.**
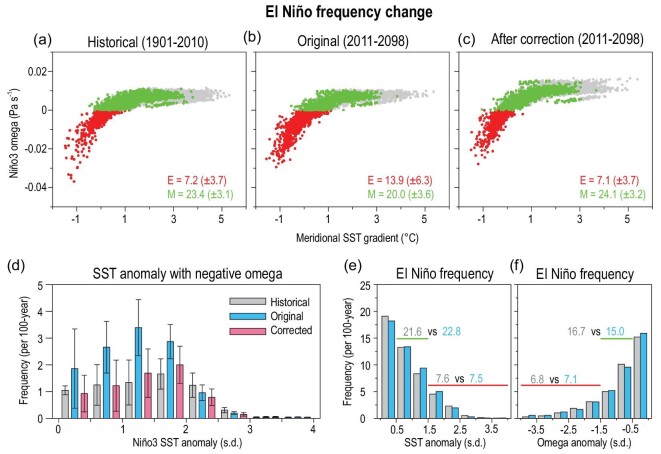
El Niño frequency change in the RCP4.5 scenario. Relation between the Niño3 omega and the north-minus-south meridional SST gradient in the eastern Pacific. Red, green and gray dots indicate extreme El Niño events (i.e. negative Niño3 omega), moderate El Niño events (i.e. positive omega but with greater than 0.5 standardized SSTA in Niño3 region), and non-El Niño years, respectively [4]. Results are based on (a) the historical simulations during 1901–2010, (b and c) the original and corrected projections of the extreme El Niño frequency in the RCP4.5 scenario during 2011–2098. All the frequencies are calculated per 100 years. (d) Histogram of the extreme El Niño frequency in each magnitude bin of the Niño3 SSTA (interval: 0.5 standard deviation). The 95% confidence interval of the extreme El Niño frequency is estimated by bootstrap test. (e) Frequency of extreme and moderate El Niño, defined as the standardized Niño3 SSTA in boreal winter being greater than 1.5 (red line) and being 0.5–1.5 standard deviation (green line), respectively. The frequency of the extreme and moderate El Niño events (per 100 years) in the historical simulations and future projections are represented by gray and blue Arabic numbers, respectively. (f) As in (e), but for the results defined by Niño3 omega anomaly in boreal winter.

In agreement with the strong relation between the extreme El Niño frequency change and Niño3 omega mean-state change, our results indicate that the former would be rectified after removing the total net impacts of the 13 common biases on the projected mean-state change (Fig. 5, Methods).

The original projections in the RCP4.5 scenario indicate that the extreme El Niño frequency would increase by 93% in the future (i.e. 13.9 vs. 7.2 events per 100 years) [2–4], concurrent with a slightly decreased moderate El Niño frequency (Fig. 5a and b). In stark contrast, after the correction, the frequency of both the extreme and moderate El Niño would barely change (Fig. 5c). Similarly, the original projections of 152% increase of the extreme El Niño frequency in the RCP8.5 scenario (13.1 vs. 5.2 events per 100 years) would also be reduced to an insignificant 17% increase (6.1 vs. 5.2 events per 100 years) (Fig. S5a–c). Results based on 88 years (1923–2010) and, recently, 30 years (1981–2010) are similar (Table S2).

In the original projections with an El Niño-like warming change, the stronger warming in the eastern Pacific would weaken the Walker circulation and the subsidence in the east, and thus favor moderate SST anomalies there to trigger deep convection (Fig. 5d) [2–4]. However, the frequency of the extreme and moderate El Niño defined by either the SST or omega anomaly in the Niño3 region would barely change in the future (Fig. 5e and f). After the correction, the SST change would display a weak La Niña-like warming in the Pacific. The occurrence of the extreme El Niño defined by deep convection [4] would still require a strong SST anomaly forcing in the eastern Pacific, similar to that in the past century (Fig. 5d and Fig. S3e). Results based on the RCP8.5 scenario are similar (Fig. S5d–f).

## DISCUSSION

In contrast to previous studies [2–4], our findings indicate that the future extreme El Niño frequency is over-projected due to systematic biases in CMIP5 models. After removing total net impacts of 13 well-recognized biases on the mean-state projections, the MME projection displays a La Niña-like change, opposite to the original El Niño-like change [2–4,24,26]. While the El Niño definition itself is probably uncertain in a warmer climate, the extreme El Niño frequency, defined both by the total values [2–4] after the bias-correction and by the original anomalies, would barely change in the future. This helps reduce the uncertainty/debate with regard to the projected future change.

Our results are consistent with the common notion that many increased extreme events are largely induced by mean-state changes in the past and future [33]. Nevertheless, the statistical method for correcting future extreme El Niño frequency might not be perfect; in particular, possible impacts of complex interactions between ENSO and mean-state change on the future extreme El Niño change remain to be explored [7–9,13,23,34,35]. In addition, the significant correlations among many of the 13 processes/mean-states indicate that fundamental processes (such as cloud physics) may be commonly misrepresented in CMIP5 models. This requires much advanced climate models that can accurately reproduce these important processes. Before reaching this stage, the results here can provide useful information on whether the model biases may impact future projections and how large the impacts of the biases could possibly be. This will certainly help advance model development in future.

## MATERIALS AND METHODS

### Materials

We examine the impacts of 13 common biases based on 30 models that participated in CMIP5. To minimize possible influences of internal decadal variabilities, we use a 110-year period (1901–2010) to estimate the model biases. Correspondingly, the maximum period (2011–2098) is selected to estimate future projections. We also examine the results based on 88 years (2011–2098 vs. 1923–2010) and 30 years (2069–2098 vs. 1981–2010) with high-quality observations. The historical runs mostly end in 2005, so outputs from 2006 to 2010 are from the RCP4.5 scenario. Future projections are represented by RCP4.5 and RCP8.5 scenarios from 2011 to 2098. Monthly mean fields of SST, precipitation, total cloud cover, near-surface zonal wind and omega, averaged from 500 hPa to 0 hPa, are used.

We utilize the average of three or four re-analysis datasets to minimize the uncertainty. Specifically, re-analysis datasets used in this study include monthly SST from the National Oceanic and Atmospheric Administration (NOAA) Extended Reconstructed SST V5 [36], the UK Met Office Hadley Centre's HadISST data [37], Centennial *in situ* Observation-Based Estimates (COBE) data [38], and ERA-20C [39]. Total cloud cover datasets are from ERA-20CM [39], NOAA-20CRV2 [40] and NOAA-20CRV3 [41]. Surface zonal wind datasets are from the NOAA-20CRV2 [40], NOAA-20CRV3 [41] and ERA-20C [39], respectively. All model outputs and re-analysis datasets are interpolated to a 1° × 1° grid.

The MME mean is defined as the equal weight average of the 30 models. Note that some CMIP5 models have more than one member. For these models, each member is calculated independently, and then a simple average is used to get the ensemble mean of that model. This approach is used throughout the analysis.

### Methods

SST, precipitation and omega changes at each latitude and longitude grid between the future climatology (2011–2098) and the present-day climatology (1901–2010) are divided by the global mean SST change in each model to account for the different sensitivity of each model to global warming.

Please refer to the Supplementary Data for the details of the ‘model grouping’ method, the ‘emergent constraint’ method [42,43] and the ‘multiple emergent constraint’ method [44].

The projected monthly fields (i.e. SST, surface wind, precipitation and omega) during 2011–2098 can be divided into their long-term climatology (i.e. $\bar{A}$) and their anomaly relative to the $\bar{A}$. Here, we correct the $\bar{A}$ to examine the impacts of the models’ common biases on the extreme El Niño frequency change in the future:

$$\begin{equation*}{\rm{\ }}{\bar{A}^*} = \bar{A}\ - {\rm{TI}} \times \Delta \overline {SST,}\end{equation*}$$

where ${\rm{TI\ }}$ represents the total net impacts of the 13 common biases on the projected future changes in SST, surface wind, precipitation and omega, respectively. $\Delta \overline {SST} $ represents the global mean SST change. ${\bar{A}^*}$ represents the long-term climatology after the correction.

## Supplementary Material

nwab056_Supplemental_File
