# Supplementary material for: Over-projected Pacific warming and extreme El Niño frequency due to CMIP5 common biases
Source: Natl Sci Rev. 2021 Apr 6;8(10):nwab056. doi: 10.1093/nsr/nwab056 (PMC8566187; doi:10.1093/nsr/nwab056)
Supplement: nwab056_Supplemental_File [file nwab056_supplemental_file.docx]

**Supplementary Information**

**Over-Projected Pacific Warming and Extreme El Niño Frequency due to CMIP5 Common Biases**

Tao Tang^1,2,†^, Jing-Jia Luo^1,2,*, †^, Ke Peng^1,2^, Li Qi^2^, Shaolei Tang^1^

^1^ Institute for Climate and Application Research (ICAR), Nanjing University of Information Science and Technology, Nanjing 210044, China

^2^ Key Laboratory of Meteorological Disaster of Ministry of Education/Joint International Research Laboratory of Climate and Environment Change/Collaborative Innovation Center on Forecast and Evaluation of Meteorological Disasters, Nanjing University of Information Science and Technology, Nanjing 210044, China

* Corresponding author. **Emails:**  jjluo@nuist.edu.cn; [jingjia_luo@hotmail.com](mailto:jingjia_luo@hotmail.com).

† Equally contributed to this work.

**This PDF file includes:**

Supplementary text

Figures S1 to S5

Tables S1 to S4

**Supplementary Information**

A few methods have been recently proposed to reduce the influences of model biases on future projections. One is model weighting, namely, better models are more trusted or weighted [45]. However, this method is not suitable for correcting the impacts of models’ common biases, because the biases may still exist in the better models. Another popular method is “emergent constraint” [42], which was widely used to reduce the uncertainty of the CMIP5 future projections, including polar warming [43]. It was also used to correct the impacts of cold tongue mean-state bias on the projection of future SST change [26]. However, this method was usually applied to investigate the impact of a single model bias. The comprehensive influences of multiple common biases on the projection of future tropical Pacific SST change and consequently the extreme El Niño frequency change are still unclear.

Here, we examine individual and total net impacts of the biases in simulating 13 well-recognized processes/mean-states in the CMIP5 models (Table S1, S3). For each process/mean-state, we analyze its potential impact on the Pacific SST and surface wind change projection using a composite method. We rank the models according to their performance in simulating the present-day observations, and then the models are divided into red, gray, and blue groups, which represents good, neutral, and bad models, respectively. The red and blue groups have the same number of models, but may vary among the different processes.

To ensure the red and blue groups are significantly different from each other, the criterion for the model grouping is that the difference between the last model in the red group and the first model in the blue group is larger than one standard deviation (s.d.) of the spread of all the models.

The emergent constraint method is used to calculate the impacts of a single common bias [26,42,43]. In addition, the multiple regression method is used to calculate the total impacts of the 13 processes/mean-states by removing the inter-dependent influences (i.e., multi-collinearity) among the 13 processes/mean-states (Table S4) [44].

**The emergent constraint method.** The emergent constraint method is used to calculate the impacts of a single common bias. The difference between individual model simulation and the MME in each examined process/mean-state can be represented by HMD

$HMD= ms-ms (MME)$ (1)

The difference between individual model projected change and the MME projected change of fields (i.e. SST, winds, precipitation, and omega) in future can be represented by FMD

$FMD= fc-fc (MME)$ (2)

The emergent constraint method can be seen as a linear regression between$\mathrm{FMD}$ and $\mathrm{HMD}$

$FMD=a\times HMD+b$ (3)

Note that for each process/mean-state, FMD is a three-dimensional value (i.e. model, latitude, and longitude), while HMD is one-dimensional (i.e. model).

The impacts of a common bias on the MME projection of future change calculated by the emergent constraint (IEC) are

$IEC=a\times common bias+b$ (4)

where *a* is the regression coefficient and *b* is the intercept.

**The multiple regression method.** Using the 13 processes/mean-states as predictors, we can reconstruct the projected changes of the extreme El Niño frequency in future based on a simple multiple linear regression method.

FC=$\sum_{1}^{13} (a_{M}\times\mathrm{ms}_{M})+b$ (5)

where FC denotes the reconstructed frequency change, $\mathrm{ms}_{M}$ and $a_{M}$ denote each process/mean-state and its regression coefficient, $\sum_{1}^{13}$indicates the summation of the 13 processes/mean-states, and $b$ denotes the intercept.

Similarly, the relation between FMD and HMD is

FMD_M_=$\sum_{1}^{13} (a_{M}\times\mathrm{HMD}_{M})+b$ (6)

where M denotes each process/mean-state. Therefore, the total net impacts of the 13 common biases (TI) are

$TI=\sum_{1}^{13} (a_{M}\times{common bias}_{M})+b$ (7)

**Statistical significance tests.** We have applied various statistical tests to assess the significance of the results. To test whether the extreme El Niño frequency change in the future relative to that in the historical simulations is significant (Fig. 5a-d, Fig. S5a-d), we adopt the bootstrap test [2], in which El Niño events in each model are selected 10000 times randomly. The 95% confidence intervals are estimated. We use two-tailed Student *t*-test (Figs. 2b, 3b, d, f, h, j, 5e-f, Figs. S2, S4 b, d, f, h, j, l, n, S5e-f, Table S2 and S4) and the statistical significance of the 90% confidence, unless stated otherwise.

To show uncertainty in the original (corrected) MME projection of the SST, surface wind, precipitation, and omega change, we use stippling at each grid where more than 75% of the models show the same sign as the MME (Fig. 4, Fig. S1). The stippling in Fig. S3a-c indicates where more than 75% of the CMIP5 models show a larger variance of the SST anomaly than the tropical Pacific regionally-averaged variance of the SST anomaly.

**Data availability**

NOAA ERSST v5, [https://www.ncdc.noaa.gov/data-access/marineocean-data/extended-reconstructed sea-surface-temperature-ersst-v5](https://www.ncdc.noaa.gov/data-access/marineocean-data/extended-reconstructed%20sea-surface-temperature-ersst-v5);

NOAA-CIRES 20th Century Reanalysis version 2c, <https://www.esrl.noaa.gov/psd/data/gridded/data.20thC_ReanV2c.html>;

COBE SST, <https://psl.noaa.gov/data/gridded/data.cobe2.html>;

ERA-20CM, <https://apps.ecmwf.int/datasets/data/era20cm-edmm/levtype=sfc/>;

ERA-20C, <https://apps.ecmwf.int/datasets/data/era20c-moda/levtype=sfc/type=an/>;

NOAA-20C3V, <https://psl.noaa.gov/data/gridded/data.20thC_ReanV3.html>;

CMIP5 database, <http://www.ipcc-data.org/sim/gcm_monthly/AR5/Reference-Archive.html>.

1. Raeisaenen J, Ruokolainen R and Ylhaeisi J. Weighting of model results for improving best estimates of climate change. *Clim Dyn* 2010; **35**: 407-22.


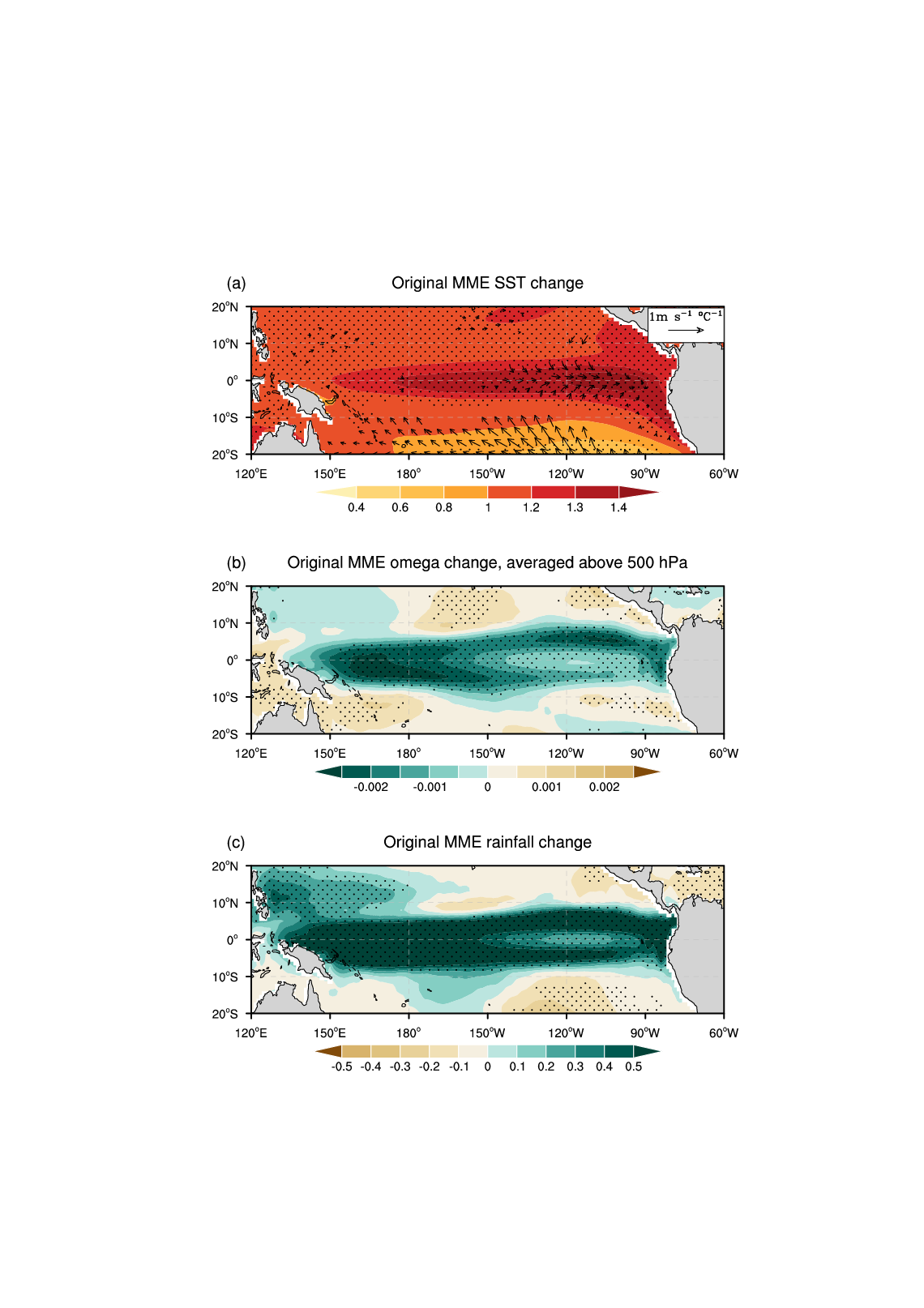


**Figure S1. Original MME projections of future change in the tropical Pacific.** The changes are represented by differences between the future period (2011-2098) and the present period (1901-2010). Before obtaining the MME, the difference in each model is divided by the model projected global mean SST change. (a) SST change (°C per degree of global SST warming), (b) omega (upward negative) averaged from 500 to 0 hPa (Pa s^-1^ ºC^-1^), and (c) precipitation (mm day^-1^ ºC^-1^). Stippling and vector indicate more than 75% of the CMIP5 models agree with the sign of MME.


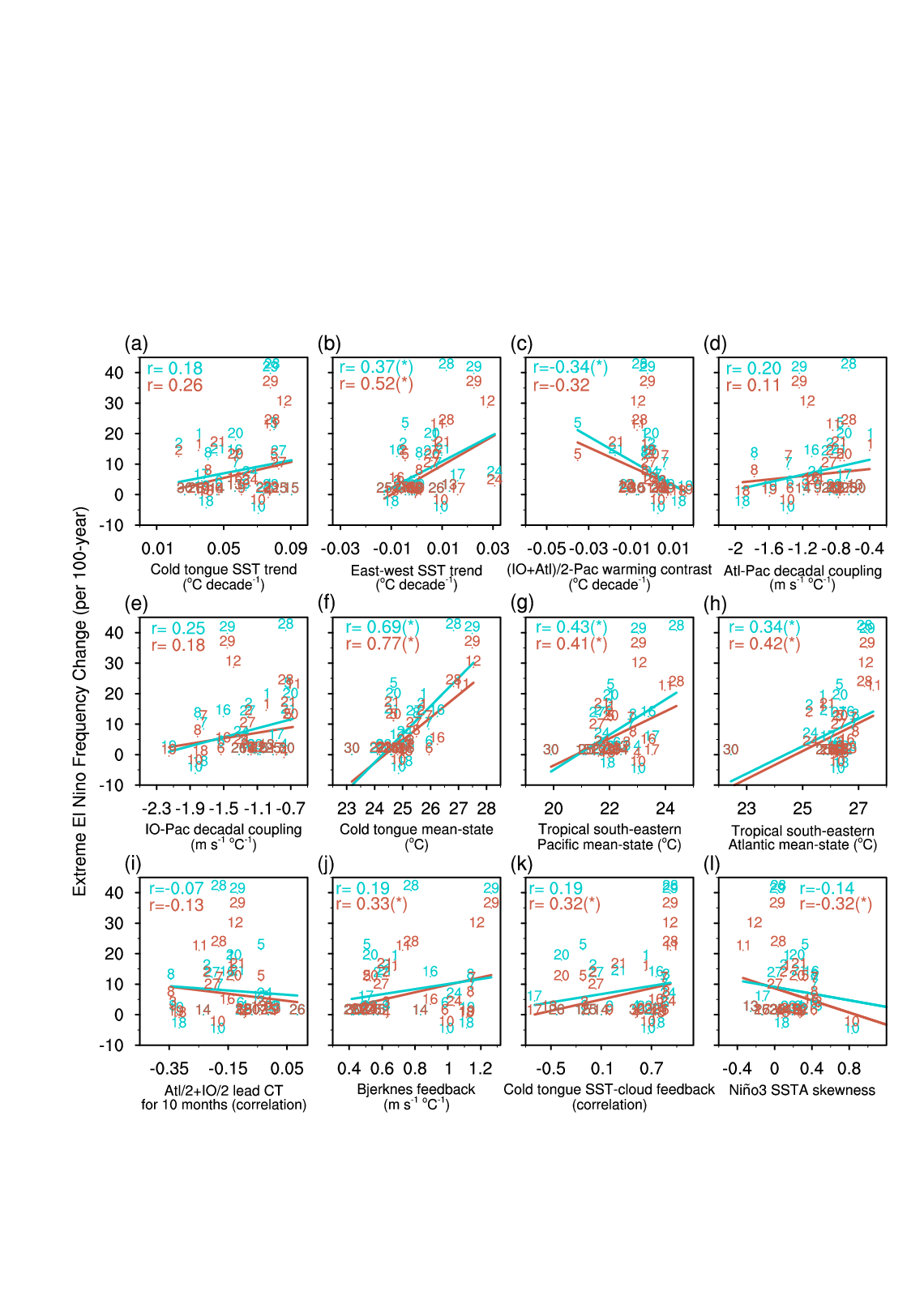


**Figure S2. Inter-model correlations between the extreme El Niño frequency change and 12 processes/mean-states in CMIP5 models.** As in Figure 1a, but for the inter-model relations between the extreme El Niño frequency change (Y axis) and the present-day simulation (X axis) of (a) centennial Cold Tongue (CT) SST trend, centennial trends of (b) east-west Pacific SST gradient and (c) inter-basin (i.e., Atl/2+IO/2-Pac) SST warming contrast, decadal coupling of (d) the tropical Atlantic and Pacific and (e) the tropical Indian Ocean (IO) and Pacific, (f) CT mean-state, (g) tropical southeastern Pacific SST mean-state, (h) tropical southeastern Atlantic SST mean-state, (i) tropical IO and Atlantic SST anomaly lead the CT SST anomaly for 10-month, (j) the Bjerknes feedback, (k) CT SST-cloud feedback, and (l) Niño3 SSTA skewness, respectively. The correlation coefficients with an asterisk are significant at 90% confidence level according to the Student *t*-test. Definitions of these processes/mean-sates are given in Table S1.


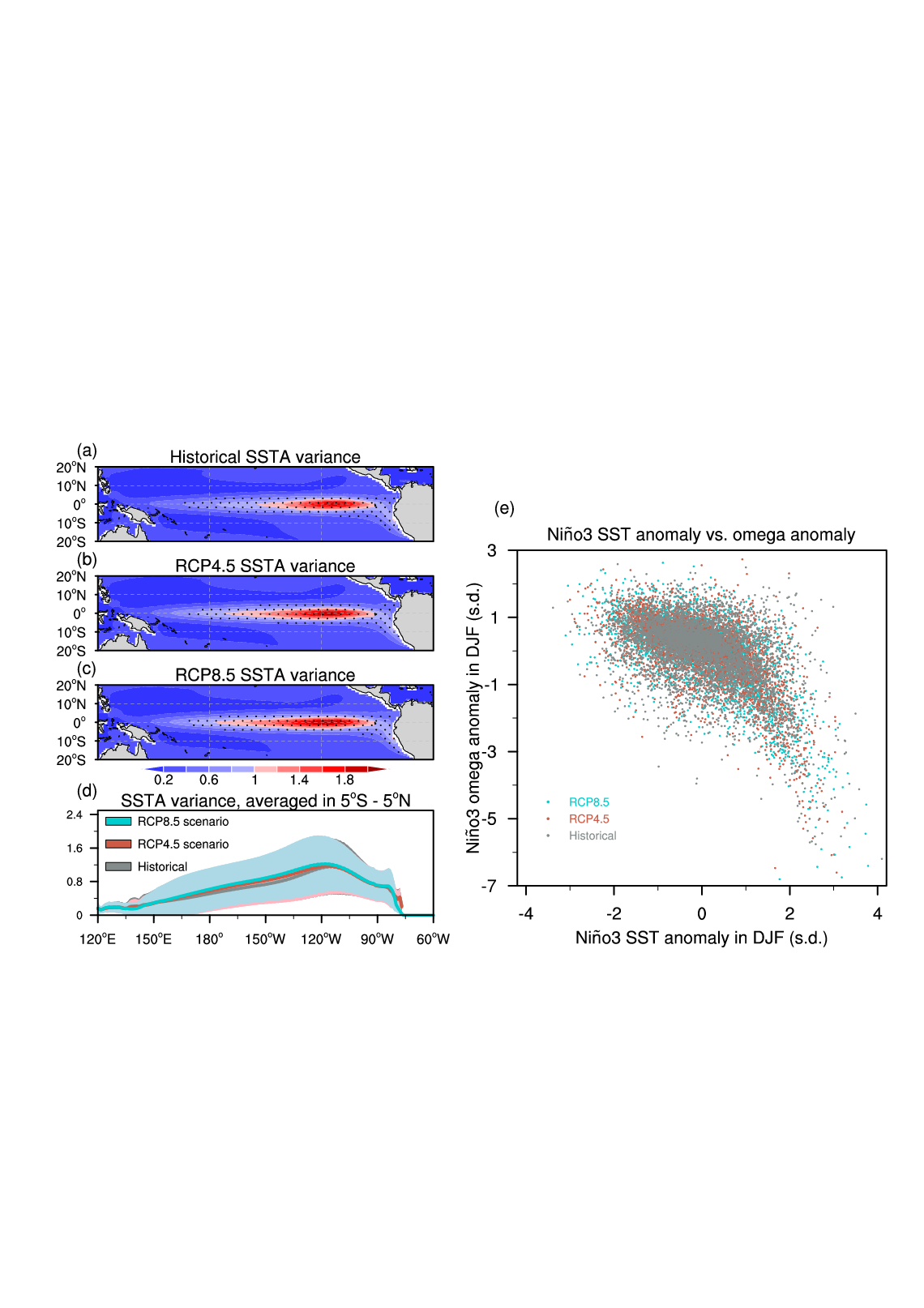


**Figure S3. Variance of monthly SSTAs and the relation between Niño3 SST anomaly and Niño3 omega anomaly.** Variance of quadratically detrended SSTA is first calculated for each CMIP5 model independently; variances are then averaged to represent the MME result. (a) MME SSTA variance in historical period (1901-2010), and (b-c) MME SSTA variance in RCP4.5 and RCP8.5 scenarios during 2011-2098. Stippling indicates the areas where more than 75% of the CMIP5 models project larger SSTA variance than the tropical Pacific regionally-averaged variance (unit: ºC^2^). (d) Monthly SSTA variance averaged along the equatorial Pacific (5ºS-5ºN). Gray, coral, and cyan colors indicate the results based on the historical simulation, RCP4.5 and RCP8.5 scenario, respectively. The solid lines represent MME, while the colored shading represents ±1 standard deviation for each simulation/scenario. (e) Scatter plot of the quadratically detrended SST (X axis) and omega anomalies (Y axis) in Niño3 region in boreal winter. Gray, coral, and cyan dots indicate historical simulation, RCP4.5 and RCP8.5 scenario, respectively.


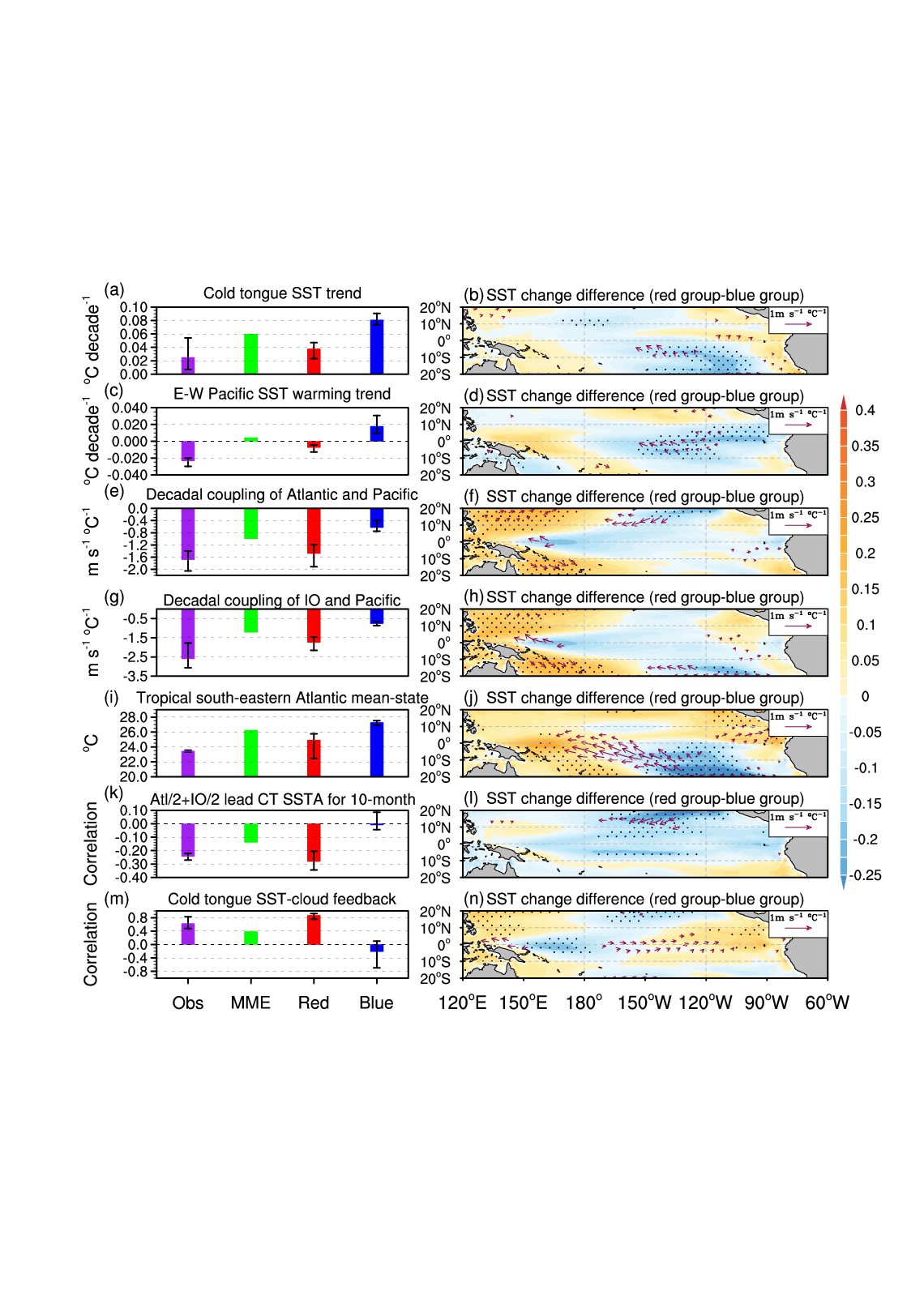


**Figure S4. Seven biases in simulating the present-day climate and their inter-group differences on the future projection.** As in Fig. 2a, b, but for the results of the CT SST trend, east-west Pacific SST warming trend, decadal coupling of the Atlantic and Pacific, decadal coupling of the IO and Pacific, tropical southeastern Atlantic mean-state, Atl/2+IO/2 lead CT SSTA for 10-month, and CT SST-cloud feedback, respectively (Table S1).


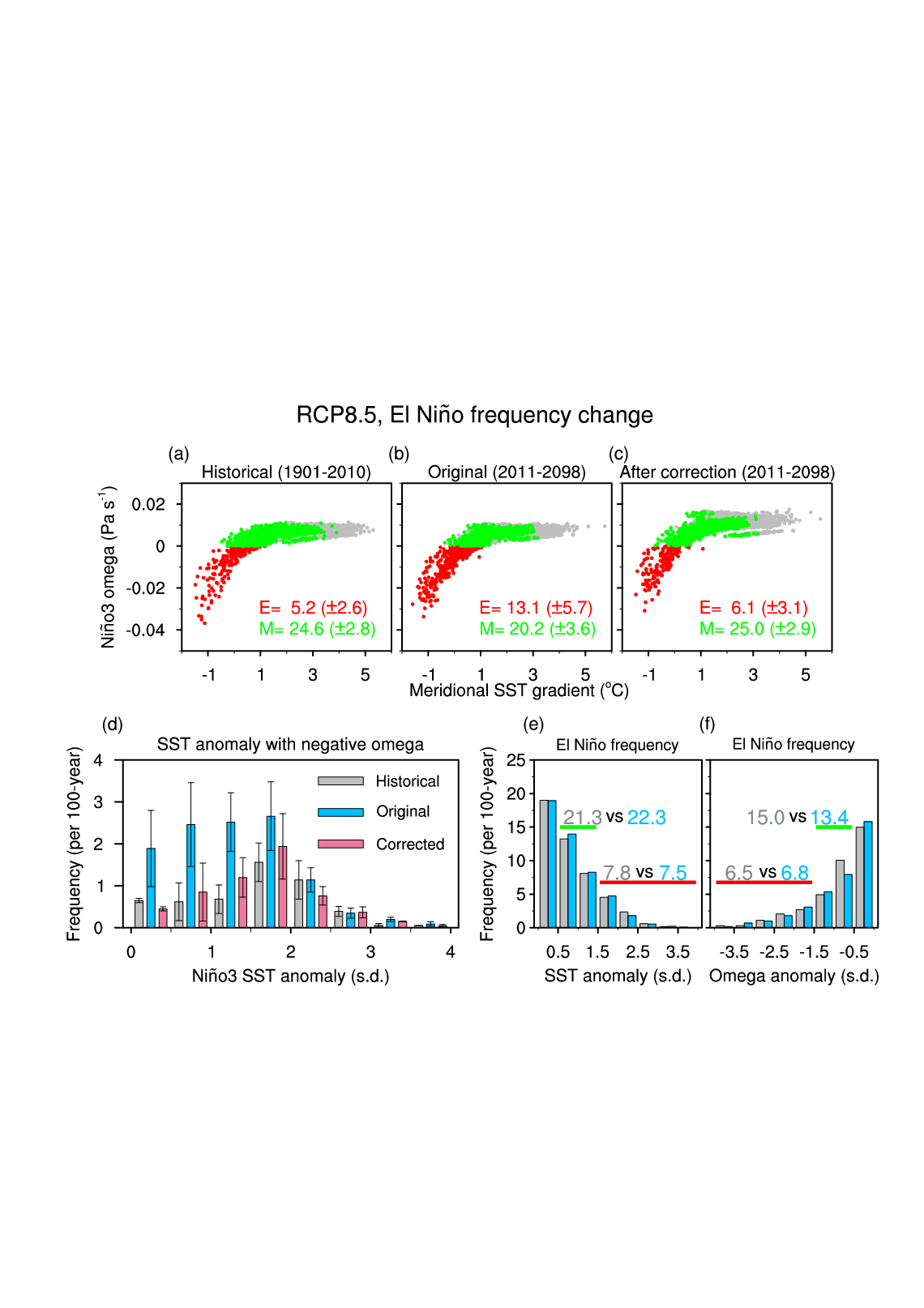


**Figure S5. El Niño frequency change in RCP8.5 scenarios.** As in Fig. 5, but for the frequency of El Niño events in RCP8.5 scenario based on 28 CMIP5 models (Table S3).

| **Number** | **Processes/mean-states** | **Definitions of the processes/mean-states** |
| --- | --- | --- |
| 1 | Zonal wind trend | Trend of 1000-hPa zonal wind averaged in (150ºE-150ºW, 10ºS-10ºN)^14^ |
| 2 | Cold tongue (CT) SST trend | SST trend averaged in Pacific CT area (170ºW-90ºW, 5ºS-5ºN)^14^ |
| 3 | East-west Pacific SST warming trend | SST trend in the eastern Pacific (180º-90ºW, 10ºS-10ºN) minus that in the western Pacific (120ºE-180º, 10ºS-10ºN)^14^ |
| 4 | Atl/2+IO/2-Pac SST warming trend | The mean of the SST trend in tropical Atlantic (Atl, 70ºW-20ºE, 10ºS-10ºN) and Indian Ocean (IO, 40ºE-120ºE, 10ºS-10ºN) minus the trend in the tropical Pacific (120ºE-90ºW, 10ºS-10ºN)^14^ |
| 5 | Decadal coupling of the tropical Atlantic and Pacific | Linear regression coefficient of the 10-year running trend of the Paciﬁc zonal wind (150ºE-150ºW, 10°S-10°N) onto 10-year running mean SST climatology difference between the tropical Paciﬁc and Atlantic^12,18^ |
| 6 | Decadal coupling of the tropical IO and Pacific | Linear regression coefficient of the 10-year running trend of the Paciﬁc zonal wind onto10-year running mean SST climatology difference between the tropical Paciﬁc and IO^15^ |
| 7 | CT mean-state | SST climatology in the CT area^14,22,24,26^ |
| 8 | Tropical southeastern Pacific mean-state | SST climatology averaged in (100ºW-70ºW, 30ºS-5ºS)^14,22^ |
| 9 | Tropical southeastern Atlantic mean-state | SST climatology averaged in (0-20ºE, 20ºS-0)^16,22^; results base on the tropical Atlantic (20ºS-20ºN) are similar. |
| 10 | Atl/2+IO/2 lead CT SSTA for 10 months | Lead-lag correlation of the monthly SSTA in the tropical Atlantic and IO lead the CT SSTA by 10 months^14,17^ |
| 11 | Bjerknes feedback | Linear regression coefficient of the monthly Niño4 zonal wind anomaly (160ºE-150ºW, 5ºS-5ºN) onto Niño3 SSTA^32^ |
| 12 | CT SST-cloud feedback | Linear correlation coefficient between the SSTA and total cloud-cover anomaly in the CT area^14^ |
| 13 | Niño3 SSTA skewness | The skewness of 5-month running mean SST anomaly in Niño3 region (150ºW-90ºW, 5ºS-5ºN)^13^ |

**Table S1. Definitions of 13 well-recognized processes/mean-states during 1901-2010.**

The first column indicates the serial number of each process/mean-state (i.e., NO. 1 to NO. 13), the second column displays their names, and the third column gives their definitions.

|  | RCPs | 110-year  (2011-2098)  vs.  (1901-2010) | 88-year  (2011-2098)  vs.  (1923-2010) | 30-year  (2069-2098)  vs.  (1981-2010) |
| --- | --- | --- | --- | --- |
| Correlation of the extreme El Niño frequency change between the projection and the multiple linear regression model’s reconstruction by the 13 processes/mean-states | RCP4.5 | ***0.92*** | ***0.90*** | ***0.65*** |
|  | RCP8.5 | ***0.90*** | ***0.89*** | ***0.75*** |
| Extreme El Niño frequency change vs.  Niño3 omega change | RCP4.5 | ***-0.87*** | ***-0.86*** | ***-0.59*** |
|  | RCP8.5 | ***-0.71*** | ***-0.73*** | ***-0.49*** |
| Niño3 omega change  vs.  the Pacific east-west SST gradient change | RCP4.5 | ***-0.68*** | ***-0.65*** | ***-0.51*** |
|  | RCP8.5 | ***-0.69*** | ***-0.70*** | ***-0.65*** |
| The extreme El Niño frequency  (historical vs. original projection) | RCP4.5 | 7.2 (±3.7)  vs.  13.9 (±6.3) | 7.8 (±3.9)  vs.  13.9 (±6.3) | 9.6 (±5.1)  vs.  16.0 (±7.3) |
|  | RCP8.5 | 5.2 (±2.6)  vs.  13.1 (±5.7) | 5.4 (±2.7)  vs.  13.1 (±5.7) | 6.3 (±2.9)  vs.  18.4 (±8.3) |
| The extreme El Niño frequency  (historical vs. corrected projection) | RCP4.5 | 7.2 (±3.7)  vs.  7.1 (±3.7) | 7.8 (±3.9)  vs.  9.5 (±4.6) | 9.6 (±5.1)  vs.  10.0 (±4.8) |
|  | RCP8.5 | 5.2 (±2.6)  vs.  6.1 (±3.1) | 5.4 (±2.7)  vs.  7.4 (±2.8) | 6.3 (±2.9)  vs.  9.3 (±4.8) |

**Table S2. The extreme El Niño frequency changes based on three different reference periods.** The bold italic font indicates the 90% confidence level according to Student *t*-test. The 95% confidence interval of the extreme El Niño frequency is based on bootstrap test.

| Number | Model name | Historical/RCP4.5/8.5 Ensemble |
| --- | --- | --- |
| M1 | ACCESS1-0 | 1/1/1 |
| M2 | ACCESS1-3 | 1/1/1 |
| M3 | bcc-csm1-1-m | 1/1/1 |
| M4 | bcc-csm1-1 | 1/1/1 |
| M5 | MIROC-ESM | 1/1/1 |
| M6 | CMCC-CM | 1/1/1 |
| M7 | CMCC-CMS | 1/1/1 |
| M8 | CMCC-CM5 | 1/1/1 |
| M9 | GFDL-ESM2G | 1/1/1 |
| M10 | GFDL-ESM2M | 1/1/1 |
| M11 | GISS-E2-H-CC | 1/1/0 |
| M12 | GISS-E2-R-CC | 1/1/0 |
| M13 | HadGEM2-AO | 1/1/1 |
| M14 | inmcm4 | 1/1/1 |
| M15 | IPSL-CM5A-MR | 1/1/1 |
| M16 | IPSL-CM5B-LR | 1/1/1 |
| M17 | MRI-CGCM3 | 1/1/1 |
| M18 | NorESM1-M | 1/1/1 |
| M19 | NorESM1-ME | 1/1/1 |
| M20 | MIROC-ESM-CHEM | 1/1/1 |
| M21 | GFDL-CM3 | 3/3/1 |
| M22 | MPI-ESM-LR | 3/3/3 |
| M23 | MPI-ESM-MR | 3/3/1 |
| M24 | MIROC5 | 3/3/3 |
| M25 | IPSL-CM5A-LR | 4/4/4 |
| M26 | HadGEM2-ES | 4/4/1 |
| M27 | CanESM2 | 5/5/5 |
| M28 | GISS-E2-H | 5/5/1 |
| M29 | GISS-E2-R | 6/6/1 |
| M30 | CSIRO-Mk3-6-0 | 10/10/10 |
| M31 | BNU-ESM | Start from 1951 |
| M32 | CESM1-BGC | Missing uas |
| M33 | HadGEM2-CC | Missing wap |
| M34 | CESM1-CAM5 | Missing uas |
| M35 | FIO-ESM | Missing uas |
| M36 | CESM1-WACCM | Missing uas |
| M37 | CCSM4 | Missing uas |

**Table S3. CMIP5 models of historical, RCP4.5, and RCP8.5 experiments used in this study.** Note that GISS-E2-R-CC and GISS-E2-H-CC have no RCP8.5 scenario experiments.

| **No.** | **2** | **3** | **4** | **5** | **6** | **7** | **8** | **9** | **10** | **11** | **12** | **13** |
| --- | --- | --- | --- | --- | --- | --- | --- | --- | --- | --- | --- | --- |
| **1** | ***0.33*** | ***0.64*** | ***-0.59*** | 0.30 | ***0.34*** | ***0.50*** | ***0.44*** | 0.29 | ***0.32*** | 0.12 | 0.05 | -0.24 |
| **2** |  | 0.16 | -0.19 | 0.16 | 0.22 | 0.31 | 0.22 | ***0.52*** | 0.18 | 0.01 | 0.14 | 0.02 |
| **3** |  |  | -0.13 | 0.07 | 0.01 | ***0.45*** | ***0.35*** | 0.21 | 0.20 | ***0.40*** | 0.16 | 0.05 |
| **4** |  |  |  | ***-0.49*** | ***-0.52*** | 0.01 | 0.03 | 0.03 | ***-0.41*** | ***0.34*** | 0.25 | 0.20 |
| **5** |  |  |  |  | ***0.87*** | -0.21 | -0.15 | ***-0.33*** | ***0.60*** | ***-0.77*** | ***-0.45*** | ***-0.33*** |
| **6** |  |  |  |  |  | -0.18 | -0.05 | -0.22 | ***0.45*** | ***-0.74*** | ***-0.42*** | ***-0.36*** |
| **7** |  |  |  |  |  |  | ***0.69*** | ***0.66*** | ***-0.32*** | ***0.62*** | ***0.45*** | -0.20 |
| **8** |  |  |  |  |  |  |  | ***0.71*** | -0.23 | ***0.35*** | 0.23 | -0.11 |
| **9** |  |  |  |  |  |  |  |  | *-*0.27 | ***0.42*** | 0.24 | -0.13 |
| **10** |  |  |  |  |  |  |  |  |  | ***-0.49*** | ***-0.35*** | -0.08 |
| **11** |  |  |  |  |  |  |  |  |  |  | ***0.69*** | 0.31 |
| **12** |  |  |  |  |  |  |  |  |  |  |  | 0.30 |

**Table S4. CMIP5 inter-model correlations among the 13 processes/mean-states.** The CMIP5 inter-model correlations among the 13 processes/mean-states (see Table S1) are based on 30 models’ simulations. The bold *italic font* indicates the 90% confidence level according to Student *t*-test.
